# Supplementary material for: The self-organized learning of noisy environmental stimuli requires distinct phases of plasticity
Source: Netw Neurosci. 2020 Mar 1;4(1):174–99. doi: 10.1162/netn_a_00118 (PMC7055647; doi:10.1162/netn_a_00118)
Supplement: Supplementary file 1 [file netn-04-174-s001.pdf]

Krüppel, S., Tetzlaff, C. (2020). Supporting information for "The self-organized learning of noisy environmental stimuli requires distinct phases of plasticity." *Network Neuroscience*, *4*(1), 174-199. https://doi.org/10.1162/netn_a_00118

Supplementary Material for

The self-organized learning of noisy environmental stimuli requires distinct phases of plasticity

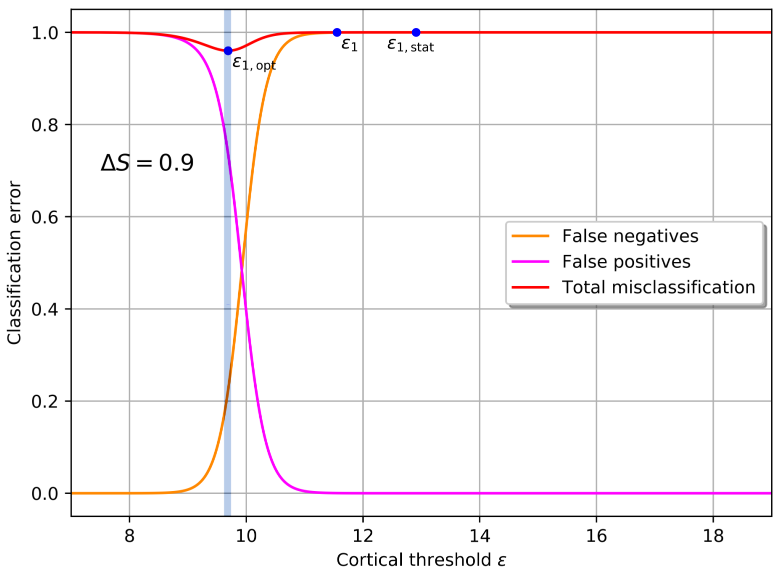

Supp. Fig. 1. Dependency of the classification error on the firing threshold for $\Delta \mathbf{S}_{\text{test}} = \mathbf{0.9}$. At high levels of noise (here $\Delta S_{\text{test}} = 0.9$), the actual value of the cortical firing threshold ε does not significantly influence the resulting classification error. This error remains close to one for all ε.

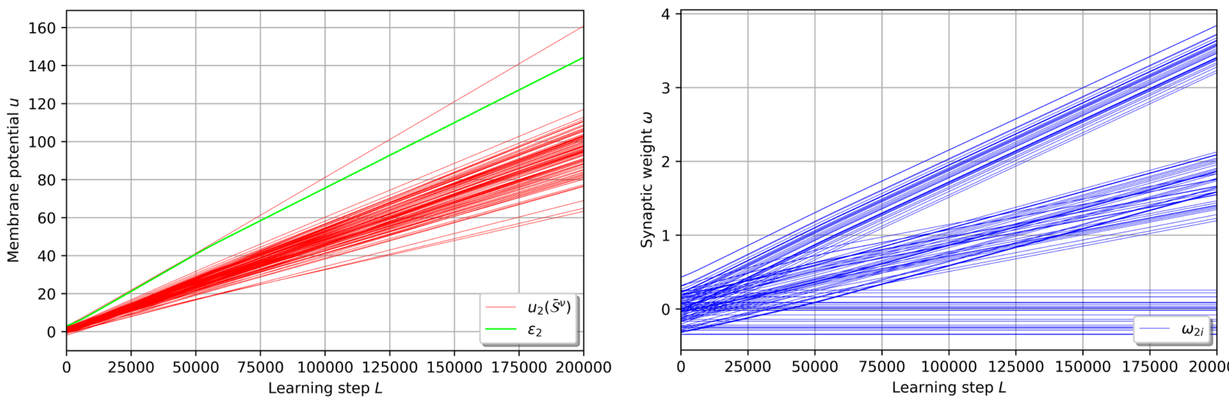

Supp. Fig. 2. Competition without homeostatic synaptic plasticity. The synaptic decay term was disabled during learning to demonstrate that competition arises from the interaction of Hebbian synaptic plasticity and homeostatic intrinsic plasticity alone. The exemplary neuron still becomes selective to only a single central pattern (left). All membrane potentials evoked by central pattern's increase. However, the firing threshold (green) is also increased such that only one stimulus pattern results in a membrane potential above the threshold. Synapses activated by this pattern are increased, but as there is no reversive synaptic decay, they keep increasing (right). Note that this simulation was done with a ten-fold smaller network.

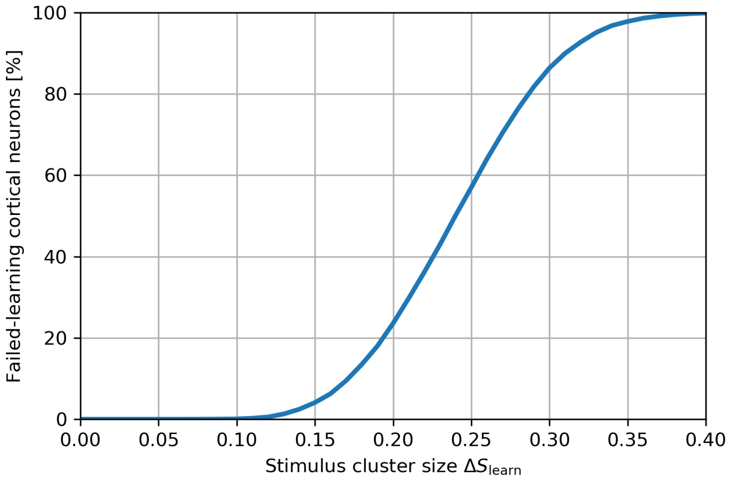

Supp. Fig. 3. The number of neurons failing to learn properly depends on the stimulus cluster size ΔS_{learn}. W.l.o.g., a neuron is classified as *failed* if no central stimulus pattern $\bar{S}^{\nu}$ yields a membrane response being above the firing threshold.

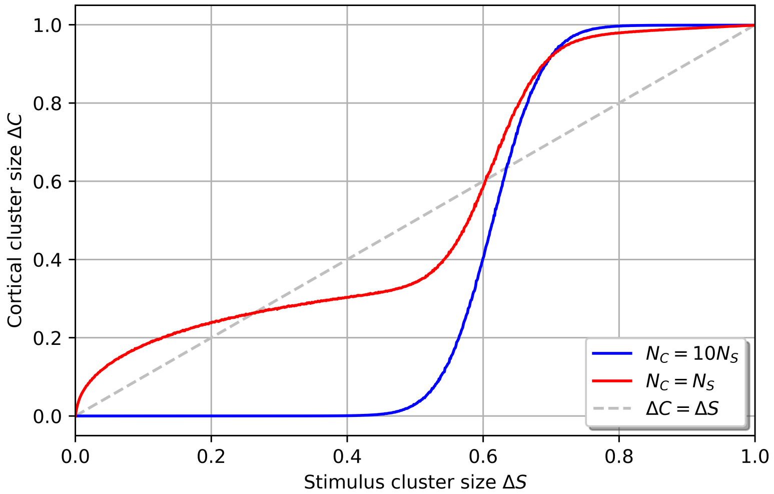

Supp. Fig. 4. Expansiveness is not a critical feature of the system. Non-expansive networks (red curve, $N_S = P = N_C = 1000$) can learn to distinguish between clusters. However, they don't reach the classification performance of an expansive network (blue curve, $N_S = P = 1000$, $N_C = 10000$). Nevertheless, this demonstrates that the results presented, in particular the proposed two-phase learning protocol, can also be generalized to non-expansive networks.
